# Activity of Zearalenone in the Porcine Intestinal Tract

**DOI:** 10.3390/molecules22010018

**Published:** 2016-12-24

**Authors:** Magdalena Gajęcka, Łukasz Zielonka, Maciej Gajęcki

**Affiliations:** 1 Department of Veterinary Prevention and Feed Hygiene, Faculty of Veterinary Medicine, University of Warmia and Mazury in Olsztyn, Oczapowskiego 13/29, 10-718 Olsztyn, Poland; mgaja@uwm.edu.pl (M.G.); gajecki@uwm.edu.pl (M.G.); 2 Department of Epizootiology, Faculty of Veterinary Medicine, University of Warmia and Mazury in Olsztyn, Oczapowskiego 13/01, 10-718 Olsztyn, Poland

**Keywords:** zearalenone, intestinal tract, gilts

## Abstract

This study demonstrates that low doses (somewhat above the No Observed Adverse Effect Level, NOAEL) of the mycoestrogen zearalenone (ZEN) and its metabolites display multispecificity towards various biological targets in gilts. The observed responses in gilts were surprising. The presence of ZEN and zearalenols (ZELs) did not evoke a response in the porcine gastrointestinal tract, which was attributed to dietary tolerance. Lymphocyte proliferation was intensified in jejunal mesenteric lymph nodes, and lymphocyte counts increased in the jejunal epithelium with time of exposure. In the distal digestive tract, fecal bacterial counts decreased, the activity of fecal bacterial enzymes and lactic acid bacteria increased, and cecal water was characterized by higher genotoxicity. The accompanying hyperestrogenism led to changes in *m*RNA activity of selected enzymes (cytochrome P450, hydroxysteroid dehydrogenases, nitric oxide synthases) and receptors (estrogen and progesterone receptors), and it stimulated post-translational modifications which play an important role in non-genomic mechanisms of signal transmission. Hyperestrogenism influences the regulation of the host’s steroid hormones (estron, estradiol and progesteron), it affects the virulence of bacterial genes encoding bacterial hydroxysteroid dehydrogenases (HSDs), and it participates in detoxification processes by slowing down intestinal activity, provoking energy deficits and promoting antiporter activity at the level of enterocytes. In most cases, hyperestrogenism fulfils all of the above roles. The results of this study indicate that low doses of ZEN alleviate inflammatory processes in the digestive system, in particular in the proximal and distal intestinal tract, and increase body weight gains in gilts.

## 1. Introduction

This article summarized the results of studies conducted in our Department in the last six years, and it makes a reference to recent scientific achievements relating to the estrogenic mycotoxin ZEN. Our studies have focused on ZEN’s multispecificity towards selected cells and tissues of the porcine gastrointestinal tract [1,2]. It is a novel approach in the existing body of research [3], which so far has targeted mainly tissues with large clusters of estrogen receptors (ERs).

Plant materials are often contaminated by *Fusarium* mycotoxins, not always at high concentrations [4,5]. Plant materials are widely used in the production of food and feed, and cereal products account for a large part of human and animal diets [6]. For this reason, knowledge relating to pharmacokinetic processes involving mycotoxins, examined in preclinical studies, is required to guarantee the safety of food and feed products. Zearalenone and its metabolites, α-zearalenol (α-ZEL) and β-zearalenol (β-ZEL), are among the most ubiquitous mycotoxins in animal feeds.

Zearalenone and its metabolites are characterized by structural similarity to estradiol, and they disrupt reproductive functions in animals [7]. Alfa-zearalenol is the predominant ZEN metabolite in pigs, whereas in other animal species, including broilers, cattle and sheep, the main metabolic product of ZEN is β-ZEL, with much lower metabolic activity [8,9]. The activity of ZEN is determined by biotransformation processes in plants [10], animals [11] and the immune status [12,13] of the reproductive system [14,15] (due to changes in steroid hormone levels during sexual maturation, reproductive cycle or pregnancy) and the gastrointestinal tract [16] of animals exposed by this mycotoxin.

Many authors have demonstrated that ZEN is immunotoxic at low concentrations [5,17,18,19]. Under exposure to low doses of this mycotoxin, target cells undergo many changes (multispecificity [2]) during vital metabolic processes such as proliferation, differentiation, apoptosis and synthesis of biologically active molecules [20,21,22]. Research into ZEN has been intensified in recent years, however, the mycotoxin’s effect on the intestinal barrier, intestinal contents and potential relationships between exposure to ZEN and intestinal inflammations [23] or apparent digestibility [24] have been rarely investigated. Most studies examined the jejunum and duodenum [21,22,25].

### 1.1. Mode of Action in Toxicology

The progress in toxicology requires continuous improvement in analytical techniques, including new methods of organizing and interpreting research results. New techniques for evaluating toxicological and chemical risks, including those associated with undesirable substances, are required to fully understand the obtained results. A robust knowledge of biological pathways that underlie toxicity and tolerance to factors disrupting toxicological processes is needed [26]. Contemporary research in mold toxicity focuses on the dose-response relationship and the Mode of Action (MOA) of a given chemical substance, and the results could be extrapolated to humans [27,28,29,30].

The mode of action documents functional and anatomical changes at cellular level provoked by exposure to a given substance. It is one of the key elements of risk assessment for classifying carcinogenic compounds and systemic toxins, and it is also used to extrapolate low doses of undesirable substances whose molecular mechanisms have not yet been fully elucidated. Molecular mechanisms play a significant role in biotransformation processes of various tissues, and they often determine which early biotransformation changes constitute Key Events (KEs). This approach is used to determine whether a given substance or organic compound inhibits or stimulates the toxicity of a metabolite or the parent compound [29].

Recent research has demonstrated extensive variations in toxicity standards, and it revealed that numerous animal species can be used in toxicity research for the needs of human medicine. The approach based on MOA could limit uncertainty in risk assessment in many areas. The use of the porcine model in preclinical studies is one of the factors that minimize the associated risk. Pigs are a highly useful model for translational research and clinical studies which are vital for improving human health. Pigs and humans have many anatomical and physiological similarities. Both are omnivores and are characterized by similar physiological and biotransformation processes [31].

The presence of small amounts of mycotoxins with endocrine activity [32], including mycoestrogens such as ZEN, in food and feed can provoke different KEs than high doses in initial stages of exposure [15]. Earlier KEs can induce a cascade of other events, which are resolved without any intervention, are not accompanied by clinical symptoms [33] or can be the first symptom of an undesirable effect. Estrogens, including estrone (E_1_) estradiol (E_2_) and estriol (E_3_), have both short-term and long-term uterotrophic effects. Progesterone (P_4_) is an antagonist of estrogens, and it reduces the sensitivity of target tissues to estrogens. Estrogens induce the synthesis of progesterone receptors, therefore, they can be used to inhibit the estrogen response [8,29,34]. However, according to Simon et al. [29], endocrinological studies often produce surprising results that testify to numerous defects in MOA which could be rectified over time.

### 1.2. Low-Dose Hypothesis

The dose of the administered mycotoxin is an important consideration in toxicological research. The symptoms and health consequences of high doses of most mycotoxins have been extensively investigated [35]. The health (toxicological) effects of low mycotoxin doses became apparent in the last decade. They are associated with hormesis [36,37], a biological phenomenon whereby exposure to doses below the NOAEL [38] induces disease without clinical symptoms (subclinical state) [15,39] or delivers beneficial effects for the host in various stages of life [40]. The dose-response relationship was put into question by the low-dose hypothesis, in particular with regard to hormonally active chemicals [41] such as ZEN and its metabolites. Those compounds can act as Endocrine Disruptors (EDs) when ingested in small doses. Due to the ambiguous nature of the dose-response relationship, the clinical symptoms and laboratory results associated with exposure to high mycotoxin doses cannot be directly extrapolated to low doses [42]. The low-dose hypothesis, which postulates that the lowest dose of a mycotoxin may provoke different results than expected, is garnering increasing interest in biomedicine. For this reason, a compound’s mode of action has to be thoroughly investigated to understand its effects on living organisms [43].

### 1.3. Interactions as an Element of MOA

The interactions between mycotoxins and the interactions between mycotoxins and physiological processes in tissues, cells and the exposed microorganisms also pose a significant problem [44,45,46,47]. To date, studies of animals exposed to mycotoxins have examined only the influence of the diet on morphological changes (proliferation, crypt depth, size of villi) and the animals’ health [21,22].

Some authors described the impact of mycotoxins on proliferation processes in intestinal cells and on bowel morphology. In pigs, chickens and mice, T-2 toxin induced necrotic changes in the intestinal epithelium and in jejunal and ileal crypts [48]. The transport of antigens from the intestinal lumen during inflammations and allergic reactions [49] is intensified by higher permeability of the intestinal barrier caused by epithelial damage and by enhanced phagocytosis/transcytosis of enterocytes. A similar scenario is observed when enterocytes are exposed to low doses of deoxynivalenol (DON), ochratoxin and patulin. The transport of commensal bacteria across the intestinal epithelium is increased without any changes in intestinal permeability [23]. Higher doses of the above mycotoxins increase intestinal permeability and mediate the transport of molecules across cells (transcellular pathway) or between the cells (paracellular pathway) [50].

## 2. Activity of ZEN

Mycotoxins are absorbed [51], metabolized [52] and distributed [53] mainly via the gastrointestinal system which is most vulnerable to the toxic effects of those substances, including ZEN and ZELs [54].

### 2.1. Multispecificity and Activity of Estrogens

**Figure 1:**
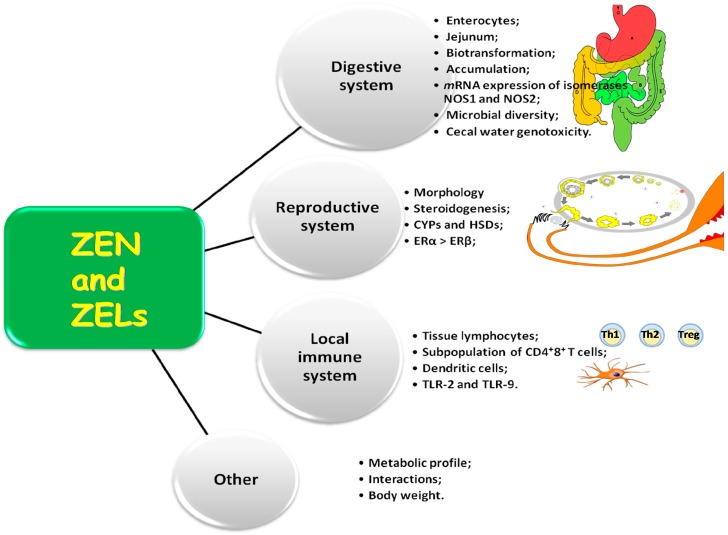
Activity of zearalenone.

In this study, the biological activity of ZEN and ZELs was investigated in view of their multispecificity (Figure 1). The multispecificity concept is used in many contexts in molecular and cellular biology to denote that one molecule of a mycoestrogen, including ZEN, can have more than one biological target or can act as a substrate for many biotransformed chemicals in tissues and cells [15]. This concept is also used to characterize estrogens and: (i) specific enzymes such as cytochromes P450 (CYPs) and hydroxysteroid dehydrogenases (HSDs) which biotrasnform (catalytic promiscuity) or are influenced by substrates (substrate promiscuity), such as ZEN and ZELs [55]; and (ii) the local immune system [2,18]. This study was also conducted on the assumption that one estrogen molecule should have high affinity for more than 250 target cells [56].

This study was undertaken to demonstrate that the statement "one ligand is one binding site" is largely outdated [2,57]. Zearalenone and ZELs can act as both ligands and/or substrates for specific biologically active substances [8,23,58]. An active substance can bind with various substrates, including ZEN and ZELs, in different conformations (non-identical arrangements produced by the rotation of backbone atoms around one or more single bonds), and it can catalyze various conversions (changes as the result of which products gain new properties) in different directions [59]. The above expands the spectrum of ligands, which can be characterized by somewhat different biological action [16,59,60].

**Figure 2:**
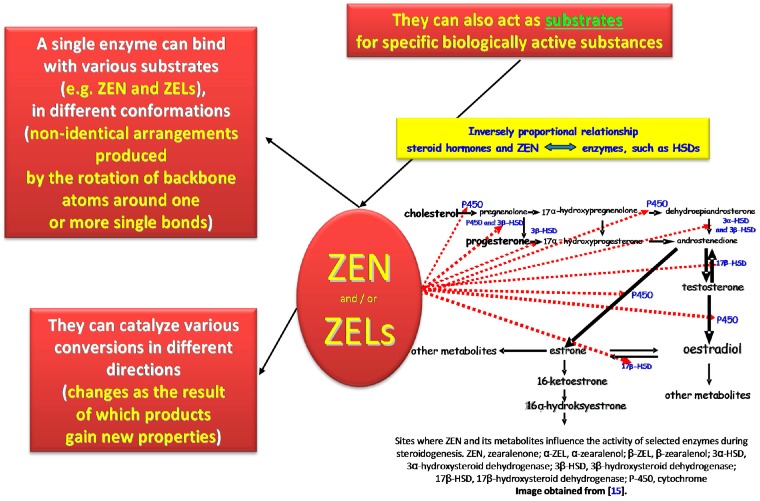
The statement “one ligand is one binding site” is not always true.

The presented study also addresses the fact that the lock and key model for estrogens is incomplete [57] (Figure 2). ERs bind with more than one ligand (including E_2_, ZEN and ZELs), and they metabolize a wide range of substrates, where only selected substrates are loosely connected to the key molecule. Estrogens bind with and modulate the activity of circulating proteins with binding properties: (i) nuclear and membrane receptors; (ii) immunocompetent cells; and (iii) other sites that have not yet been discovered. The degree of uncertainty, a characteristic feature of biological systems, continues to increase, which indicates that future research into the immunomodulatory properties of enzymes [23], their ligands, receptors [61,62] and substrates [15] requires highly accurate methods and tools.

The capacity of biological research is limited when we assume that ZEN and ZELs target only a single receptor. Zearalenone and ZELs can target more than one receptor, and their combined activity (e.g., ZEN and ZELs + E_2_ = hyperestrogenism) can serve similar and/or related purposes. Zearalenone and ZELs can influence gene expression [8,16], stabilize *m*RNA [60], stimulate translation, support interactions with receptors [16,59], influence immunocompetent cells in the reproductive system and digestive tract [18,21,47], and, together with E_2_, they can simultaneously induce all of the above processes.

At the molecular level, estrogens have been initially described as transcription regulators [60]. Exposure to environmental stressors [63] first leads to intracellular trafficking and initiation of intracrine activity, whereas other types of activity (transcription and translation) are stimulated later [59] Transcription can be a fallback position, whereas trafficking, which is more difficult to analyse, can produce responses that are most significant from the biological point of view [2].

#### 2.1.1. Interactions between Estrogens and Enzymes

The above information suggests that steroidogenesis involves numerous CYPs and HSDs (Figure 2), protein groups that participate in electron transport during oxidative phosphorylation and oxidize toxic compounds and metabolites to detoxify tissues in nearly all living organisms. Those enzymes are responsible for the synthesis of estrogens from cholesterol or their conversion [64]. The key estrogens are E_1_, E_2_ and E_3_. In the first stage, cholesterol is converted to androstenedione (Figure 2), after which E_1_ synthesizes aromatases. Alternatively, androstenedione is converted to testosterone by 17β-HSD, and to E_2_ by aromatases, although this explanation seems doubtful from the physiological point of view [65,66]. Specific differences in estrogen synthesis and conversion are observed between tissues. For example, during pregnancy, most mammals produce E_3_ in the placenta through the conversion of dehydroepiandrosterone (DHEA) and DHEA sulfate from fetal and maternal adrenal glands. The activity of various estrogens is also regulated by sulphate conjugation. The fourth estrogen, estetrol (E_4_), is synthesized in the fetal liver [67]. DHEA is one of the most widely distributed steroids in the body. This intermediate metabolite is produced by adrenal glands and gonads, and it participates in the biosynthesis of androgens and estrogens [66,68].

E_2_ is an endogenous estrogen that is most widely distributed in female vertebrates of reproductive age. Estrogen levels in fertile females range from 30 to 400 pg/mL. The hormone is synthesized mainly in granulosa cells of ovarian follicles and corpus luteum in prepubertal and adult females. Estrogen is produced locally in peripheral tissues for paracrine regulation of estrogen function. The hormone is secreted by the respective cell, and it acts upon target cells via interstitial fluid without the involvement of the circulatory system. Local production of small amounts of estrogen plays a very important role [69]. Depending on the species, estrogen synthesis levels can fluctuate occasionally (in frogs in response to rain), twice a week (in many marine animals), once a month (in pigs and humans), every six months (in cattle) or twice a year (in elephants and dogs). Males synthesize small amounts of E_2_ in Leydig cells in adrenal glands, brain and adipose tissue [69].

The fact that androgens are converted to estrogens in the presence of aromatases is an important consideration. Aromatases decrease the rate of estrogen biosynthesis, and the level of aromatase expression (inversely proportional) points to fluctuations in estrogen production. Estrogen levels can increase (hyperestrogenism) due to the compensation of endogenous and exogenous estrogens, which can be accompanied by an increase in cortisol levels and a decrease in aromatase activity. Those processes mark the beginning of adrenarche [70] during which adrenal androgens are produced before or in early stages of sexual maturation [71]. Snawder and Lipscomb [72] and Lathe et al. [2] reported that during biotransformation, the expression of this group of enzymes can be determined by the bioavailability of specific substrates, such as estrogens and hormone-like substances, or interactions with substances present in feed, including mycoestrogens such as ZEN and ZELs [73]. Aromatase activity is influenced by the presence of ZEN and ZELs in feed [25]. Aromatases are also found in various tissues outside the reproductive system and adrenal glands. They are expressed in muscles, liver, blood, heart, hair follicles, adipose tissue, brain, bones [74] and the gastrointestinal tract [16,45,60], which suggests that estrogen plays various non-reproductive roles. Interestingly, the host’s hormones can influence the virulence of bacterial genes (through bacterial HSDs), which implies that ZEN can be an etiological agent of chronic multifactorial disorders such as inflammation or cancer [75]. The results of published studies also indicate that some microorganisms, including gut bacteria, demonstrate HSD activity, among them *Comamonas testosteronii*, *Alcaligenes*, *Treponema denticola* and *Escherichia coli* [75,76]. Intestinal bacteria from patients diagnosed with colon cancer were capable of degrading estrogens, which suggests the presence of a close relationship between microbial metabolism and the etiology of chronic diseases [77,78]. The question that remains to be answered is whether there are any similarities between reproductive system bacteria and gut bacteria.

#### 2.1.2. Participation of Selected CYPs and HSDs

The metabolism of environmental estrogens, including mycoestrogens, is often regarded as a detoxification process which lowers the concentrations of the parent substance (in this case, ZEN), but also leads to the production of new compounds that can be by far more toxic (e.g., α-ZEL) than the parent substance [79]. In vitro and in vivo studies have demonstrated that ZEN reduces the activity of many P450scc cytochrome enzymes that participate in steroidogenesis as well as HSDs, including 3β-HSD, 17β-HSD and their isomers (Figure 2), that convert pregnenolone to P_4_ or E_1_ to E_2_ [34,66]. Those enzymes can protect the body against dangerous and undesirable substances while exacerbating their toxic effects (e.g., ZEN and ZELs) through the catalyzed processes [80].

Gajęcka et al. [34] demonstrated that biotransformation of ZEN induced a seven-fold increase in the number of *m*RNA transcripts in bitches, but only for 3β-HSD. At the same time, *m*RNA levels increased two-fold for the CYPscc gene, which probably blocked final reactions in steroidogenesis. A significant increase in P_4_ concentrations was reported during exposure to ZEN doses above NOAEL values. Those processes would be very interesting to observe in gilts.

**Figure 3:**
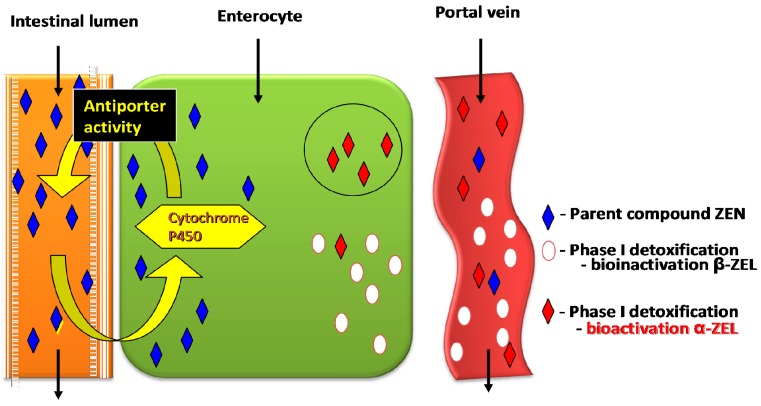
Antiporter activity in enterocytes.

Low activity of phase 1 detoxification enzymes could be attributed to low energy levels [47,81], which is also crucial for antiporter activity (Figure 3) in enterocytes [73]. The balance between detoxification phases 1 and 2 can be thus disrupted, subject to ZEN dose. As a result, metabolites such as α-ZEL and β-ZEL enter the body and modify the activity of steroidogenesis enzymes (3β-HSD and 17β-HSD), depending on the dose of the substrate. Alpha-ZEL can also destabilize proliferation processes in young animals at tissue level [16,82], which is of particular importance in farm animals [13].

The described situation is accompanied by atypical values of the carryover factor in peripheral blood in successive weeks of exposure [47]. This can be attributed to the fact that mycoestrogens inhibit enzyme activity by lowering, for example, P_4_ concentrations to IC_50_ values. Therefore, mycoestrogens influence not only the activity of steroid metabolizing enzymes and, consequently, the activity of endogenous estrogens and androgens in their synthesis sites, but also the activity of P_4_ in peripheral tissues [82,83,84]. The accompanying drop in α-ZEL levels can be attributed to: (i) an increase in the concentration of the substrate (ZEN) available to 3β-HSD; or (ii) inhibition of the activity of 3β-HSD oxidase due to substrate excess [85].

#### 2.1.3. The Role of ERs in Estrogen Signaling Pathways

Estrogen levels are strictly controlled by ERs in growing animals. Various types of disorders are observed when estrogen signaling is disrupted due to ER defects or sub-optimal estrogen levels. Defective estrogen signaling can also result from exposure to exogenous estrogens, including ZEN and ZELs. The basic functions of those exogenous estrogens in developing vertebrates should be researched in greater detail to facilitate the identification of potential risks [69]. Estrogens, including E_2_ and mycoestrogens, easily permeate cell membranes, and ERs are implicated in their cellular activity (estrogen signals). Mammals have two ligands activating transcription factors that bind estrogens and/or mycoestrogens as competitive substrates that modulate the activity of enzymes responsible for estrogen biosynthesis [16,86]), Those ligands are encoded by separate ERα (ESR1/ERα) and ERβ (ESR2/ERβ) genes [60]. ERs are composed of several domains that are important for hormone, phytoestrogen, mycoestrogen and DNA binding, dimer creation and transcription activation [87]. The DNA-binding domain is highly conserved for both ER types and species, which suggests that they can bind with similar *cis*-regulatory elements in chromatin. In humans, ER gene expression is specific and determined by receptor sub-type and the type of tissues or cells where ERs are present. Both can produce receptor homodimers and heterodimers. E_2_ binds with ERα and ERβ with similar affinity, but in the estrogen response element, ERβ is a much weaker transcription activator than ERα, and ERβ has no effect in ERE-API elements, which are responsible for the proliferative response to E_2_. This could suggest that ERβ modulates ERα activity in cells by inhibiting estrogen-dependent proliferation and promoting apoptosis [88], when both receptors are co-expressed. The problem is that ERβ is expressed without ERα in many cells, and in those cells, ERβ plays roles that are independent of ERα. Such cells are found in selected neurons of the central nervous system [89], microglia [90], prostate [91] and colonic endothelium [92].

E_2_ activates ERα and ERβ with similar affinity despite only 56% similarity in their ligand-binding domains [91,93]. By contrast, ZEN’s affinity for ER in target cells and tissues accounts for only 1%–10% in comparison with E_2_ [85,94]. The activity of ZEN and ZELs in target tissues is also influenced by the type of ER. In human cells, ZEN demonstrates greater activity and affinity for ERα than ERβ, and it is a strong agonist of ERβ [95]. According to other authors, ZEN’s in vitro interactions with ERβ in human cells have a mixed character subject to its concentration: when ZEN interacts more actively with ERβ than ERα, α-ZEL is more likely to bind with ERα than ERβ, and vice versa [94]. The degree (%) of binding was always much lower that during ER binding with E_2_. ERs can also be activated by post-translational modification (when genetic information contained in DNA is completely translated into specific protein structures, depending on the location of amino acids in the polypeptide chain), which influences non-genomic signal transduction [96], including by ZEN [97,98].

### 2.2. Morphometry of the Duodenum and Jejunum

The tunica mucosa of the gastrointestinal tract is exposed to much higher concentrations of the ingested mycotoxins than any other structure in the body. Therefore, special attention should be paid to the effects of these compounds on the function and structure of the duodenum and jejunum [25].

In a study by Lewczuk et al. [22], per os administration of low doses of ZEN to gilts did not induce significant morphometric changes in duodenal mucosa, a sensitive indicator of undesirable substances in feed. The influence of ZEN on duodenal morphology was investigated in greater detail by estimating the ratio of villus height to depth crypt, a highly sensitive measure of epithelial homeostasis. The above ratio decreases when cell loss is not compensated by cell proliferation [99], which was not observed in the cited study. Submucosal thickness increased, and it was accompanied by diffuse proliferation of Brunner’s glands. Proliferative processes could be attributed to adaptive or defense mechanisms against ZEN. The epithelium was not infiltrated by lymphocytes, which probably resulted from a high number of lymphocytes and plasma cells in the lamina propria. According to the cited authors, the noted results can be attributed to the presence of ERs in lymphocytes and macrophages [100,101].

Przybylska-Gornowicz et al. [21] reported an increase in the number of goblet cells in the epithelium of intestinal villi. The above was accompanied by an increase in the number of lymphocytes in the epithelial layer of villi in the initial period of exposure, and an increase in the number of plasma cells in the lamina propria towards the end of the experiment. The cited authors suggested that low doses of ZEN stimulate the immune system [19] by directly acting upon plasma cells [102].

### 2.3. The Carryover Factor and ERs

The Carryover Factor (CF) in the gastrointestinal tract of gilts exposed to low doses (former NOAEL value of 40 µg/kg BW) has never been quantified. The only study addressing this problem was conducted by Zielonka et al. [47]. According to initial estimates of exposure, low doses provoke completely different responses than those observed under exposure to high doses of mycotoxins. The main difference is that low doses induce subclinical states without recognizable clinical symptoms of disease. Weakly expressed changes (stimulatory or compensatory effect [103]) are also observed at tissue or cellular level [21,22].

Most *Fusarium* mycotoxins are absorbed predominantly in the proximal small intestine [73] due to considerable physiological differences between intestinal segments. Mucus glycoproteins are least abundant in the duodenum and jejunum, which contributes the movement of digesta across intestinal walls [51] and increases its availability. Most carbohydrates are simultaneously absorbed in the proximal part of the small intestine [104], which significantly contributes to the absorption, accumulation and, probably, biotransformation of mycotoxins in enterocytes [81,105]. Published data relating to those processes and the involved intestinal segments are incomplete and often contradictory. In vitro studies revealed that ZEN was absorbed in 51% [106] to 55% [42] in the small intestine. The role played by different intestinal segments in this process has not been determined.

The highest accumulation of ZEN (%) was noted in the small intestine during initial exposure. On the remaining dates, ZEN was also accumulated in the duodenum and descending colon. The pattern of ZEN accumulation differed completely from the carryover percentage of DON which was evenly accumulated during the period of exposure [44]. According to Zielonka et al. [47], duodenum is vital for the absorption, but not biotransformation of ZEN. Similar observations were made in our study where ZEN metabolites were not detected in the duodenum of gilts administered the pure parent compound per os. The degree of accumulation was estimated based on the value of CF which was highest in the duodenum and jejunum in the first three weeks of exposure. On the last two dates, the highest CF values were noted in the duodenum and descending colon. Our results indicate that in prepubertal gilts, ZEN is accumulated mostly in the duodenum, jejunum and descending colon [25].

In gilts exposed to the parent compound only, ZEN was not biotransformed or was biotransformed in small amounts in the digestive tract and intestinal tissues [47]. Toxicological and chemical analyses did not reveal the presence of α-ZEL or β-ZEL in the examined animals. The accumulation of ZEN in intestinal tissues began already in the first week of exposure, and it proceeded at a much higher rate in comparison with the accumulation of DON [44]. The highest concentration of DON was observed in the jejunum only in the third and fourth week of exposure. At the same time, plasma concentrations of ZEN and α-ZEL (unpublished data) point to low levels of ZEN in the peripheral blood of gilts in the first 28 days of the experiment. This could suggest that in the initial period of exposure, ZEN was biotransformed in the blood, and it was distributed to estrogen-sensitive tissues (reproductive system, bone marrow, central nervous system) in successive weeks [13,107].

According to other authors, ZEN is generally degraded by the porcine microbiota, but only in the distal colon, whereas biotransformation activity is not noted in the proximal segment of the intestinal tract [108]. Somewhat similar results were reported by Piotrowska et al. [45] who noted that long-term exposure to low doses of ZEN only had a detrimental effect on Aerobic Mesophilic Bacteria (AMB) in the descending colon. The presence of ZEN metabolites was not observed in the distal colon.

The above observations were indirectly confirmed by a study of gilts [109], which demonstrated the multidirectional effects of ZEN. In the cited study, ZEN inhibited *m*RNA expression of the gene controlling constitutional isomer NOS-1 and inducible isomer NOS-2. In the most general terms, exposure to the mycotoxin slowed down bowel peristalsis [110] and contributed to antiporter activity (Figure 3) in the jejunal wall [111]. The above can be probably attributed to slower movement of digesta through the intestines, which prolongs the period of contact between digesta and intestinal walls. This, in turn, can increase the accumulation of ZEN in the initial period of exposure and inhibit cell proliferation in various systems, including during apoptosis [48] processes which are slowed down [109,112]. An in vitro study by Chen et al. [113] demonstrated that low doses of ZEN can also stimulate autophagy. According to the cited authors, this defensive mechanism protects the body against the toxic effects of ZEN [113]. However, autophagy can contribute to the accumulation of ZEN in the small intestine, and it can increase CF values in the initial period of exposure relative to other intestinal segments and the liver, excluding the descending colon [47]. In gilts, the administration of feed containing low doses of ZEN slows down peristalsis in the proximal colon, which decelerates proliferation and stimulates autophagy, thus protecting the body against undesirable substances ingested with feed [113].

Another hypothesis could also be postulated. Our knowledge of the changes that accompany exposure to low doses of ZEN is limited, therefore, the associated side effects are difficult to predict. This uncertainty could be linked to the dose as well as the period of exposure. Low mycotoxin doses can provoke surprising responses: (i) the presence of undesirable substances, such as mycotoxins, could be ignored, which is similar to the T-regs hypothesis [114] where regulatory T cells are not expressed when the body is exposed to a small number of infectious agents; (ii) prolonged exposure to orally administered ZEN can contribute to its accumulation in the host’s body; and (iii) a compensatory effect [103] is also possible where the activity of the examined indicators is inhibited and homeostasis is restored [42] despite ongoing exposure. The accumulation of ZEN in various intestinal segments on different experimental dates provides indirect evidence for the above hypothesis: the analyzed mycotoxin was accumulated in both the duodenum and jejunum only in the first weeks of exposure [47].

The fact that CF values increased in the duodenum and jejunum at the beginning of exposure and in the duodenum and descending colon at the end of the experiment also contributes to uncertainty. It remains to be determined whether: (i) the described situation was influenced by the expression of ERs in the upper digestive tract which regulate intestinal functions [115], which would imply that estrogen activity is specifically targeted; and (ii) whether hyperestrogenism (induced by ZEN) in pre-pubertal gilts (very low values of E_2_, [34]) leads to uncontrolled cellular proliferation, inhibition of apoptosis and a decrease in the number of cell adhesion markers in colonic crypts [116]. Scientists are divided over the significance and distribution of ERs in the mammalian digestive tract [88]. ERs are unevenly distributed across tissues: (i) ERα is found mainly in bones, mammary glands, genitourinary system, cardiovascular system and the central nervous system; whereas (ii) ERβ is localized mainly in the gastrointestinal tract. Those receptors appear to play contradictory roles in the regulation of proliferative processes and the differentiation of target tissues. Research has demonstrated that ERβ modulates the expression of ERα by inhibiting the proliferation of estrogen-dependent cells and promoting apoptosis [117].

Our knowledge about the distribution of ER-positive cells in a healthy gut continues to be limited [118]. It is assumed that ER-positive cells are located mainly in the duodenum, colon and descending colon. The above would suggest that CF peaks [47] are correlated with the expression of various ERs [59]. According to our unpublished results, feed contamination with ZEN could decrease the number and activity of ERα which are already scarce in pre-pubertal animals (ERα:ERβ ratio of 1:5). E_2_ is an endogenous ligand of ERα [91], therefore, the ERα:ERβ ratio is unlikely to be higher in gilts which are characterized by very low levels of E_2_ (±6 pg/mL) [119]). ERβ is the dominant receptor in the digestive tract [120], in particular in healthy colonic mucosa [88], and its expression can be somewhat decreased by ZEN [86]. The location of ERs inside cells and non-genomic actions of estrogen-like substances through nuclear receptor ligands are also important considerations [88,121]. Hyperestrogenism could explain the decrease in the optical density of ERs over time. It should also be noted that ZEN is a competitive substrate that modules the activity of enzymes participating in steroidogenesis at the pre-receptor level, whereas the absence of or very low concentrations of α-ZEL could be attributed to decelerated biotransformation of ZEN [59].

The discussed arguments support the following observations: (i) per os administration of a naturally occurring parent compound (ZEN) does not initiate biotransformation or initiates this process below the method detection limit; (ii) during exposure to low doses of ZEN, CF values on selected days of exposure and in selected intestinal segments can be determined by the distribution and expression of ERβ; (iii) ZEN is accumulated in the jejunum and descending colon on different days of exposure, and in the duodenum throughout the entire period of exposure.

### 2.4. Microbiota Diversity in the Intestinal Lumen

The composition of intestinal microbiota and the quantitative and qualitative stability of this ecosystem are vital determinants of health in animals [122]. Gut bacteria undergo immune and metabolic reactions, and they are closely linked with the host. Intestinal microbiota is required for digestion and protection, it stimulates the host’s immune system, controls fermentation processes and prevents colonization by pathogens [122]. Gut bacteria are used for therapeutic and preventive purposes in patients with Inflammatory Bowel Disease (IBD) [23].

Microbiota can also exert a negative influence on animals. Pathogenic microorganisms produce toxic metabolites and fecal enzymes that can lead to the formation of carcinogenic substances and the activation of procarcinogenic compounds [45,123]. The majority of research investigating bacterial effects on mycotoxins focuses on gut microbiota’s ability to eliminate toxic compounds. The mechanisms by which ZEN induces quantitative changes in intestinal microbiota have not been fully elucidated [124]. According to Piotrowska et al. [45], prolonged exposure to low doses of ZEN (40 µg/kg BW) provokes significant changes. An analysis of different microbial groups revealed an increase in the counts of Lactic Acid Bacteria (LAB) in the experimental group [45]. During the entire experiment (5 weeks), LAB counts remained above 10^9^ CFU/g, and statistically significant differences were not reported between groups, whereas DON decreased LAB counts. Those results are consistent with the published data indicating that LAB are the dominant bacteria in healthy intestines [125]. LAB deliver benefits for the host by producing short-chain fatty acids (acetic acid, butyric acid, propionic acid), amino acids, B vitamins and other antibacterial metabolites (bacteriocins) that prevent pathogens from colonizing the gastrointestinal tract and stimulate the host’s immune system [122,125,126,127]. LAB can also bind mycotoxins [128,129]. According to El-Nezami et al. [129], LAB remove mycotoxins by binding them to the bacterial cell wall.

In the cited experiment, [45], the average AMB counts in the intestinal contents of gilts exposed to ZEN decreased significantly by 1 or 2 log relative to *t* = 0 in the first week. In the following six weeks, a steady decrease was noted in AMB counts. These results indicate that prolonged exposure to low doses of ZEN (40 µg/kg BW) had a negative effect on AMB. Burel et al. [130] conducted a similar experiment on fumonisin B_1_ and demonstrated that low doses of this mycotoxin did not influence intestinal microbiota in pigs. In successive weeks of the experiment, bacterial counts in all experimental groups were somewhat lower than those given in the literature. According to Drew et al. [131], the counts of aerobic bacteria ranged from 6.56 log CFU/g to 7.15 log CFU/g in the ileum and reached 7.74 log CFU/g on average in the cecum.

In the ascending colon, the counts of fecal bacteria of the family *Enterobacteriaceae* (7.82 log CFU/g) decreased by around 2 log in the initial period of exposure and remained stable until the end of the experiment. Significant differences were not observed in *Escherichia coli* counts. A decrease in the colonic counts of *Enterobacteriaceae* fecal bacteria is undesirable because it stimulates the activity of fecal enzymes β-d-glucosidase and β-d-glucuronidase. Fecal enzymes can enhance the activity of mutagenic, carcinogenic and genotoxic substances that contribute to bowel cancer [132]. High LAB counts counteract those negative effects [133]. The LAB:*E. coli* ratio was estimated at 2, which is an indicator of healthy microbiota [125]. Streptococcus counts decreased considerably during initial exposure to ZEN, but returned to normal levels in successive weeks of exposure. Fungal counts decreased significantly by 0.75 log to 3.29 log CFU/g. The predominant fungi were *Geotrichum candidum* and *Candida glabrata* yeasts.

The isolated microorganisms were characterized by intensified metabolism of carboxylic acids which accounted for 30% of the metabolized substrates and amino acids. This action can lead to the production of toxic metabolites such as ammonia, amines, phenols and indoles. Toxic metabolites exert a negative influence of enterocytes, speed up peristalsis, contribute to diarrhea in pigs and decrease productivity [122,134].

The presented results suggest that ZEN exerts a negative effect on the counts of AMB, *Clostridium perfringens*, *E. coli* and other bacteria of the family *Enterobacteriaceae* during and after 42 days of exposure.

### 2.5. Genotoxicity of Cecal Water

Zearalenone induces a host of toxic effects in mammals [85,135,136], and its role in feces could be an interesting object of scientific inquiry. Feces are a complex mixture of dietary ingredients, therefore, fecal analysis is a non-invasive method for examining the intestinal mucosa, in particular in the distal end of the intestinal tract, which provides valuable information during dietary intervention trials [46,137]. Feces are used as a biomarker for the detection of DNA and the mutagenic activity of cecal contents [138]. For this reason, cecal water can be a biomarker of the genotoxicity of cecal and colonic contents towards colonic microbiota.

According to De Ruyck et al. [139], exposure to ZEN can contribute to the accumulation of carcinogenic substances in animal tissues, which can exert harmful effects on humans. Other authors demonstrated that intestinal epithelial cells are the first target in animals orally administered low doses of ZEN [21,22,140]. Intestinal mucosa inhibits the transfer of antigens, including undesirable substances such as ZEN, commensal bacteria and pathogens into deeper tissues [141]. In a study by Nowak et al. [46], a low dose of ZEN (40 µg/kg BW) administered *per os* increased the genotoxicity of cecal water in the distal colon in the sixth week of the experiment. The above probably resulted from the accumulation of ZEN in the porcine distal colon (in the intestinal contents and tissues) [47]. An increase in genotoxicity was also noted in the proximal colon. According to the cited authors, slow transfer of intestinal contents, in particular after exposure to ZEN, increases the risk of intestinal dysfunctions and carcinogenicity. Similar conclusions were formulated by De Ruyck et al. [139].

### 2.6. Expression of Intestinal Nitric Oxide Synthase

Gajęcka et al. [109] reported significant differences in *m*RNA expression of the genes controlling NOS-1 in selected segments of the intestinal tract and the liver, in particular in the jejunum and descending colon, in the first and sixth week of exposure, with a decreasing trend. Similar trends were observed in *m*RNA expression of the genes encoding NOS-2, but the noted values were much higher in comparison with the expression levels of the genes controlling NOS-1. Those findings point to a decrease in *m*RNA expression of both genes, which could suggest that low doses of ZEN inhibit both NOS isomers and probably decrease the production of NO. Similar trends in *m*RNA expression of the genes encoding NOS-1 and NOS-2 were observed in selected segments of the intestinal tract and liver throughout the experiment. The decrease in gene expression levels became more pronounced towards the end of the experiment in the group of animals exposed to ZEN.

The results of the cited study indicate that ZEN inhibits *m*RNA expression of the gene controlling constitutive isomer NOS-1 and inducible isomer NOS-2. This suggests that prolonged exposure to low doses of ZEN can lead to specific changes in the intestinal tract of gilts due to a decrease in the levels of NO which inhibits Non-Adrenergic Non-Cholinergic (NANC) transmitters [142]. Low concentration of NO probably accelerates esophageal, gastric and intestinal motility, inhibits gastric accommodation (relaxation, delivery, adaptation and contraction) and increases intestinal sphincter contraction, which slows down gastric emptying and the passage of digesta through the intestines [143,144].

A decrease in *m*RNA expression of the gene encoding NOS-1, which is released in the enteric nervous system, should slow down intestinal peristalsis and sphincter function. A decrease in *m*RNA expression of the gene controlling NOS-2 should lower intestinal permeability and inhibit intestinal secretion [145]. The above assumptions were formulated by directly extrapolating the symptoms associated with high doses of ZEN to low doses of the mycotoxin. However, according to Vandenberg et al. [41], this is not always the case (low-dose hypothesis) because very low doses of any tissue-modulating hormone could have different effects. This is exemplified by hormonally active undesirable substances, including ZEN which can act as a signaling molecule.

The decrease in *m*RNA expression of genes encoding both NOS isomerases, in particular in the distal colon, is interesting for two reasons. Firstly, mycotoxins have bactericidal properties, and they reduce the counts of microbial pathogens, the main proinflammatory agents that stimulate NO production [146]. Secondly, low doses of ZEN inhibit *m*RNA expression of both genes encoding NOS, which could deliver therapeutic effects [142,147] at high, but not normal concentrations of NO. Therefore, the presence of small amounts of ZEN in feed inhibits inflammatory processes in the digestive tract, in particular in the small intestine and the colon. The above could involve the retreat of NO signaling molecules in response to the stimulation of the systemic and local immune system, similar to that when regulatory T cells are induced by traditional pathogens [114] during chronic infections.

### 2.7. Lymphocyte Subpopulations in Mesenteric Blood Vessels

In gilts exposed to various doses of ZEN over a period of 4 weeks, the mycotoxin caused mucosal inflammations as well as damage to the epithelium and mucosa, which is consistent with the results documenting ZEN’s negative influence on intestinal defense [42]. The results of in vivo studies demonstrated that both low and high doses of ZEN could contribute to intestinal inflammations [148] and allergies [49].

Zielonka et al. [63] observed that the proliferative activity of immunocompetent cells (T cells and B cells) from mesenteric lymph nodes in the jejunum and ileum of female wild boars exposed to ZEN and DON in naturally contaminated feed intensifies lymphocyte proliferation. The above conclusion was supported by the results of morphological analyses [21,22] of boars exposed to ZEN only. Volumetric densities of T cells increased significantly in the jejunal epithelium towards the end of the experiment. Those results indicate that the immune system participates in the elimination of mild inflammations or that proliferative activity is somewhat intensified. Prolonged exposure to low doses of ZEN stimulates the proliferative activity of T cells in mesenteric lymph nodes in the jejunum [102,149]. The above increases lymphocyte counts (concentrations of clusters of differentiation CD4^+^ and, in particular, CD8^+^) in the porcine jejunal epithelium, as previously suggested by Maresca and Fantini [23] and Maresca [107]. The local immune system responds to low mycotoxin doses towards the end of exposure, which is consistent with the hypothesis put forward by Grenier and Applegate [42] and the compensatory effect. A highly significant decrease in the subpopulation of CD4^+^8^+^ (double positive, DP) T cells was noted in mesenteric blood vessels in the fifth week of exposure. It was accompanied by a statistically non-significant decrease in the subpopulation of CD4^−^8^+^ T cells.

The observed changes in the subpopulations of DP and CD4^−^8^+^ T cells point to a decrease in the cytotoxic activity of Tc cells, but not Th cells which stimulate B cells responsible for the humoral immune response. Those observations were confirmed in a study of pigs by Przybylska-Gornowicz et al. [21], where the number of plasma cells in the lamina propria remained constant throughout the entire period of exposure. The discussed changes in lymphocyte subpopulations were noted in peripheral blood [150]. Subclinical inflammation of the small intestine [21,22] and allergic reactions [22,49] were noted in analyses of blood samples collected from mesenteric blood vessels (article in press), a region where the presence of a chemotactic factor could be expected, and in sites characterized by changes in T cell subpopulations [25]. Double-positive T cells are transported from the blood stream to intestinal mucosa [151], which increases their volumetric density in tissues.

The above probably leads to a decrease in the percentage of DP T cells, in particular CD8^+^ cells, in mesenteric venous blood in the final weeks of exposure, and similar observations were made in samples of peripheral blood [18]. These findings suggest that the decrease in the percentage of Tc cells in total lymphocyte counts could be induced by a decrease in the subpopulations of CD4^+^8^+^ (in particular cells with cluster of differentiation CD8^+^) as well as CD4^−^8^+^ T cells.

### 2.8. Evaluation of the Metabolic Profile

There is a general scarcity of published data relating to variations in hematological and biochemical parameters of animals exposed to different doses of ZEN [152]. Such information would constitute a valuable reference for interpreting our results. The observed variations can be attributed to: (i) method of mycotoxin administration; (ii) mycotoxin dose; and/or (iii) the absorption kinetics of mycotoxins. The latter could be divided into several sub-processes, including extraction from the feed matrix, absorption, distribution, accumulation in tissues, and excretion [73]. Oral administration of pure ZEN led to an absence of its metabolites in gastrointestinal tissues [47]. Metabolites were detected only in the blood stream. The described situation can have two outcomes: (i) in pre-pubertal gilts, higher energy expenditure [42] is required for the above processes to take place, which was indirectly confirmed by blood glucose levels (decreasing trend); (ii) gilts could develop different responses to ZEN in the peripheral regions of the circulatory system (*vena cava cranialis*) where blood was sampled for metabolic tests. *Vena cava cranialis* is situated far from distal segments of the intestinal tract [45] and kidneys (excretion), organs which are directly adjacent to the gastrointestinal system [140]. As a result, a minor but statistically significant increase in selected hematological and biochemical parameters of blood sampled in the peripheral region was noted during exposure to low doses of mycotoxins when CF values were relatively low during transport [47] to the intestines (towards the end of the experiment) and the liver (highest values were noted in the first week of the experiment). Changes in hematological parameters were observed in the first three weeks of exposure (WBC, basophils) as well as in successive weeks of the experiment (Hb, Ht). Those results indirectly point to the involvement of the local immune system (digestive tract) [20,25] at the beginning of exposure, and the circulatory system [153] in successive weeks of the experiment as a compensatory effect [42]. Significant differences in blood biochemistry parameters such as glucose, total protein, iron or potassium concentrations were noted at the beginning of the study. A decrease was observed only in total protein levels, probably as a result of the compensatory effect [42].

The results of biochemical analyses [152] also revealed a decrease in WBC counts, glucose levels, enzymatic activity of alanine aminotransferase (AlAT) and alkaline phosphatase (AP), and iron concentrations. The activity levels of A1AT in gilts exposed to ZEN were higher than in the control group on nearly all analytical dates, which could be indicative of fatty liver dystrophy [154,155]. The activity of the necrotic enzyme aspartate aminotransferase (AspAT) increased at the beginning of the experiment and decreased gradually in successive weeks of exposure. Those results could be attributed to very long adaptive processes in gilts (dietary tolerance [23] or compensation [42]). Adaptive processes are particularly important during exposure to ZEN because low doses of this mycoestrogen do not induce clinical changes. The low-dose effect [41] should not be disregarded because it can contribute to carcinogenesis (metabolic syndrome) preceded by vasodilation and/or neoangiogenesis [156,157]. Similar results were reported in a study by Jiang et al. [158] where hormonal homeostasis was disrupted due to hyperestrogenism induced by endogenous and exogenous estrogens in pre-pubertal animals [41,156,159].

Both aminotransferases have been long used as indicators of liver dysfunctions in humans and animals, and they are often referred to as liver-specific enzymes. The amount of aminotransferases released into the bloodstream is proportional to the number of damaged cells, and those enzymes are histological markers of inflammation [160]. In a study by Gajęcka et al. [152], aminotransferase activity increased at the beginning of exposure. In view of the CF values [47] in different segments of the intestinal tract, it could be postulated that the negative effects of exposure in the first weeks of the experiment (biotransformation and bioavailability) were eliminated in the intestinal epithelium [161]. The above was manifested by a decrease in AlAT values, which indirectly proves that: (i) the continued decrease in the activity of both enzymes can be attributed to healing processes in the liver [155]; and (ii) liver function was reinstated [162]. Such discrepancies are noted on a daily basis (balance between catabolism and anabolism), and they neutralize the observed clinical symptoms.

Very low glucose levels were observed in the cited study [152]. According to De Angelis et al. [163], hypoglycemia can probably be attributed to the conversion of native mycotoxins (at low doses) into glycosyl derivatives. Glycosyl derivatives are produced during interactions with glucose molecules which are released from ingested feed in the small intestine, in particular in the distal fragment of the duodenum and the jejunum (proximal small intestinal segment). In those segments of the small intestine, the ingested dose of ZEN was absorbed most rapidly in 80%–90% [42]. These processes require significant amounts of energy, and they could explain the decrease in glucose levels in the peripheral blood of gilts before ZEN is carried over and accumulated in small intestinal walls, a process that could be prolonged under exposure to low doses of ZEN [47].

Significant changes in red blood cell (RBC) counts and other RBC parameters were not observed in gilts exposed to low doses of ZEN [152]. A minor increase in total protein levels was noted in the exposed animals. Low doses of ZEN and its catechol metabolites have estrogen-like properties and stimulate metabolic processes [164] (in this respect, ZEN and its metabolites resemble growth stimulators [165]). The above leads to: (i) stable and higher body weight gains; (ii) alleviation of inflammatory processes in the liver, as confirmed by a decrease in AlAT activity; and (iii) increase in total serum protein levels.

Exposure to low doses of ZEN also produces a compensatory effect [103], namely the stimulation or inhibition of enzyme activity in initial weeks of exposure, followed by a return to initial values [42] despite ongoing exposure.

The results of metabolic tests performed during exposure to low doses of ZEN can point to: (i) higher demand for oxygen in certain tissues [154]; or (ii) reduced inflammation of the gastrointestinal wall [166]. The above was partly documented by Tarasiuk [25] who reported morphometric changes indicative of local inflammation of the jejunum. The cited author observed the presence of connective tissue with immune system cells, lymphocytes and plasmocytes, only in villous stroma and in spaces between crypts, in particular at the beginning of exposure. The above findings are partially compatible with our results, and they partially validate the observations made by Gerez et al. [167].

The research findings indicate that the initial period of exposure brings a stimulatory effect which is subsequently eliminated by: (i) the compensatory effect; (ii) activation of adaptive mechanisms; (iii) excessive loss of energy and protein, which could point to higher feed efficiency; or (iv) extensive involvement in detoxification processes (biotransformation).

### 2.9. Body Weight Gains

According to Marin et al. [11], ZEN does not affect body weight, average daily gains or feed intake. In contrast, other authors found that ZEN influences body weight gains [25,154,168] and the growth of selected bodily tissues [169,170]. A healthy digestive system is a prerequisite for health in animals and humans. Contamination with mycotoxins is garnering increasing interest. Efforts are being made to improve the quality of animals feed and eliminate undesirable substances [171]. In gilts exposed to low doses of ZEN, the mycotoxin comes into direct contact with intestinal mucosa after feed ingestion [20]. Highly metabolically active cells and tissues are predominant in the healthy digestive tract of pre-pubertal animals [42]. Their presence validates the hypothesis that small amounts of endogenous and exogenous substances with steroidal properties stimulate proliferative processes [172]. In a study by Gajęcka et al. [152], body weights increased over time of exposure to low doses of ZEN, which could be attributed to dietary tolerance [23,41] or the compensatory effect [103]. Those findings suggest that low doses of ZEN stimulate body weight gains in pre-pubertal gilts.

## 3. Summary—Key Events Which Play a Major Role in Clinical States

**Figure 4:**
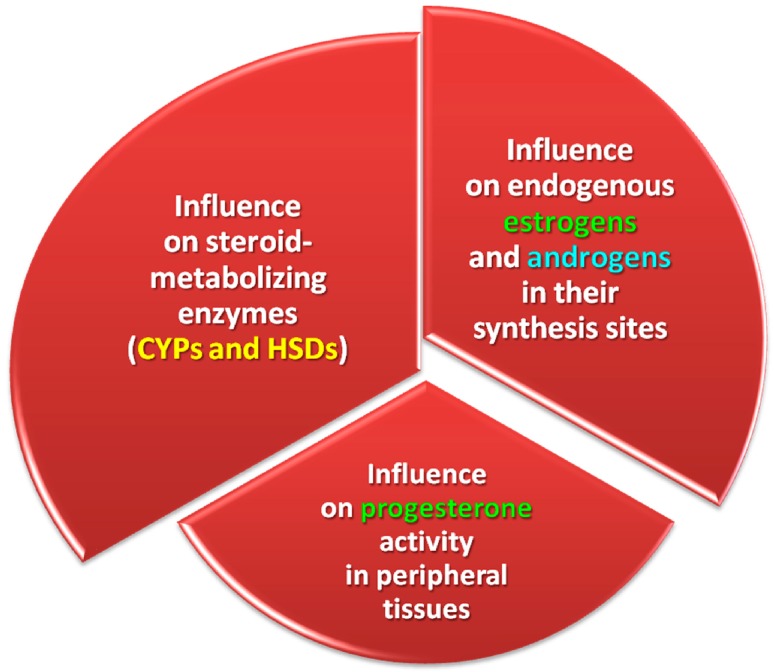
Potential key events that determine the clinical state of organisms exposed to ZEN and/or ZELs.

The biological activity of ZEN, administered orally in low doses, in the gastrointestinal tract of gilts has not been fully elucidated. Our findings and the results of published studies suggest that the following KEs are associated with exposure to ZEN (Figure 4):

- Disruptions in the proportions of ZEN and carbohydrates which are absorbed in enterocytes in the proximal segment of the intestinal tract;
- Absence or limited (below the method detection limit) biotransformation of ZEN in enterocytes after oral administration of the pure parent compound;
- Increase in CF values in selected weeks of exposure in different segments of the intestinal tract, subject to the location and expression of ERβ. In a healthy gut, ERs (mainly ERβ) are found mainly in the duodenum, jejunum and descending colon, and ERβ can modulate the expression of ERα by inhibiting the proliferation of estrogen-dependent cells and promoting apoptosis;
- Activation of ERs due to post-translational modification, which influences non-genomic signal transduction, including by ZEN;
- Defects in ERs and/or inadequate estrogen levels, which influences cellular activity (estrogen signaling pathway);
- Decrease in *m*RNA expression of both genes controlling NOS isomerases, which slows down intestinal peristalsis, stimulates sphincter contraction, decreases intestinal permeability and inhibits intestinal secretion. The above changes are noted mainly in the distal segment of the digestive tract due. Mycotoxins have bactericidal effects, and they reduce the counts of microbial pathogens, one of the key proinflammatory agents that stimulate NO production;
- Deceleration of gastrointestinal motility, which stimulates antiporter activity in jejunal walls. The above can lead to the accumulation of ZEN in intestinal walls at the beginning of exposure and the inhibition of cell proliferation, including apoptosis (loss of control over proliferation processes);
- Decrease in fecal bacterial counts in the distal colon, which stimulates fecal enzymes β-d-glucosidase and β-d-glucuronidase. Those effects are minimized when LAB concentration is higher. The genotoxicity of cecal water increased in the proximal colon;
- Stimulation of the activity of estrogen-metabolizing enzymes (CYPs and HSDs) and, consequently, endogenous estrogens and androgens at the place of their synthesis, as well as the activity of P_4_ in peripheral tissues;
- Enhanced proliferative activity of lymphocytes, which is manifested by higher volumetric density of T cells in the jejunal epithelium, an increase in the number of plasma cells in the lamina propria and the number of T cells in lymph nodes, in particular the subpopulations of CD4^+^8^+^ T cells in mesenteric venous blood and peripheral blood. The above points to a decrease in the cytotoxic activity of Tc cells, in particular CD4^+^8^+^ cells (with cluster of differentiation CD8^+^), but also CD4^−^8^+^ T cells. The local immune system participates in the elimination of subclinical inflammations and/or allergic reactions in the intestines, which is manifested by higher lymphocyte proliferation in various tissues of the digestive tract;
- A metabolic profile analysis revealed an increase in the activity of liver-specific enzymes at the beginning of exposure, followed by a decrease in successive weeks of the experiment. Those fluctuations can probably be attributed to healing processes initiated in response to liver inflammation and reinstatement of liver function. Such discrepancies are noted on a daily basis (balance between catabolism and anabolism), and they neutralize the observed clinical symptoms. The results of the metabolic profile analysis also point to higher demand for oxygen, alleviation of inflammations and considerable loss of energy and protein, which could point to higher feed efficiency, extensive involvement in detoxification or both;
- Increase in body weight gains in pre-pubertal gilts.

## References

[1] Hult K., Berglund P. (2007). Enzyme promiscuity: Mechanism and applications. Trends Biotechnol..

[2] Lathe R., Kotelevtsev Y., Mason J.I. (2015). Steroid promiscuity: Diversity of enzyme action. J. Steroid Biochem..

[3] Barton M., Meyer M.R. (2015). Nicolaus Copernicus and the rapid vascular responses to aldosterone. Trends Endocrinol. Metab..

[4] Pinton P., Accensi F., Beauchamp E., Cossalter A.-M., Callu P., Grosjean F., Oswald I.P. (2008). Ingestion of deoxynivalenol (DON) contaminated feed alters the pig vaccinal immune responses. Toxicol. Lett..

[5] Smith M.C., Madec S., Coton E., Hymery N. (2016). Natural co-occurrence of mycotoxins in foods and feeds and their in vitro combined toxicological effects. Toxins.

[6] Stuper-Szablewska K., Szablewski T., Buszko M., Perkowski J. (2016). Changes in contents of trichothecenes during commercial grain milling. LWT Food Sci. Technol..

[7] Wielogórska E., Elliott C.T., Danaher M., Connolly L. (2014). Validation and application of a reporter gene assay for the determination of estrogenic endocrine disruptor activity in milk. Food Chem. Toxicol..

[8] Gajęcka M., Zielonka Ł., Dąbrowski M., Mróz M., Gajęcki M. (2013). The effect of low doses of zearalenone and its metabolites on progesterone and 17β-estradiol concentrations in blood and body weights of pre-pubertal female Beagle dogs. Toxicon.

[9] Flores-Flores M.E., Lizarraga E., de Cerain A.L., González-Peñas E. (2015). Presence of mycotoxins in animal milk: A review. Food Control.

[10] Broekaert N., Devreese M., de Baere S., de Backer P., Croubels S. (2015). Modified *Fusarium* mycotoxins unmasked: From occurrence in cereals to animal and human excretion. Food Chem. Toxicol..

[11] Marin D.E., Pistol G.C., Neagoe I.V., Calin L., Taranu I. (2013). Effects of zearalenone on oxidative stress and inflammation in weanling piglets. Food Chem. Toxicol..

[12] Martin L.M., Wood K.M., McEwen P.L., Smith T.K., Mandell I.B., Yannikouris A., Swanson K.C. (2010). Effects of feeding corn naturally contaminated with *Fusarium* mycotoxins and/or a modified yeast cell wall extract on the performance, immunity and carcass characteristics of grain-fed veal calves. Anim. Feed Sci. Technol..

[13] Dunbar B., Patel M., Fahey J., Wira C. (2012). Endocrine control of mucosal immunity in the female reproductive tract: Impact of environmental disruptors. Mol. Cell. Endocrinol..

[14] Alm H., Brüssow K.-P., Torner H., Vanselow J., Tomek W., Dänicke S., Tiemann U. (2006). Influence of *Fusarium*-toxin contaminated feed on initial quality and meiotic competence of gilt oocytes. Reprod. Toxicol..

[15] Gajęcka M., Zielonka Ł., Gajęcki M. (2015). The effect of low monotonic doses of zearalenone on selected reproductive tissues in pre-pubertal female dogs—A review. Molecules.

[16] Gajęcki M. (2013). The Effect of Experimentally Induced Fusarium Mycotoxicosis on Selected Diagnostic and Morphological Parameters of the Porcine Digestive Tract.

[17] Escrivá L., Font G., Manyes L. (2015). In vivo toxicity studies of fusarium mycotoxins in the last decade: A review. Food Chem. Toxicol..

[18] Dąbrowski M., Obremski K., Gajęcka M., Gajęcki M., Zielonka Ł. (2016). Changes in the subpopulations of porcine peripheral blood lymphocytes induced by exposure to low doses of zearalenone (ZEN) and deoxynivalenol (DON). Molecules.

[19] Pierron A., Alassane-Kpembi I., Oswald I.P. (2016). Impact of mycotoxin on immune response and consequences for pig health. Anim. Nutr..

[20] Taranu I., Braicu C., Marin D.E., Pistol G.C., Motiu M., Balacescu L., Neagoe I.B., Burlacu R. (2015). Exposure to zearalenone mycotoxin alters in vitro porcine intestinal epithelial cells by differential gene expression. Toxicol. Lett..

[21] Przybylska-Gornowicz B., Tarasiuk M., Lewczuk B., Prusik M., Ziółkowska N., Zielonka Ł., Gajęcki M., Gajęcka M. (2015). The effects of low doses of two *Fusarium* toxins, zearalenone and deoxynivalenol, on the pig jejunum. A light and electron microscopic study. Toxins.

[22] Lewczuk B., Przybylska-Gornowicz B., Gajęcka M., Targońska K., Ziółkowska N., Prusik M., Gajęcki M. (2016). Histological structure of duodenum in gilts receiving low doses of zearalenone and deoxynivalenol in feed. Exp. Toxicol. Pathol..

[23] Maresca M., Fantini J. (2010). Some food-associated mycotoxins as potential risk factors in humans predisposed to chronic intestinal inflammatory diseases. Toxicon.

[24] Joa H., Konga C., Song M., Kim B.G. (2016). Effects of dietary deoxynivalenol and zearalenone on apparent ileal digestibility of amino acids in growing pigs. Anim. Feed Sci. Technol..

[25] Tarasiuk M. (2015). The Effect of Low Doses of Zearalenone and Deoxynivalenol on the Jejunal Mucosa, Metabolic Profile and Body Weight of Pre-Pubertal Gilts. Ph.D. Thesis.

[26] Hartung T., McBride M. (2011). Food for thought … on mapping the human toxome. ALTEX.

[27] Embry M.R., Bachman A.N., Bell D.R., Boobis A.R., Cohen S.M., Dellarco M., Dewhurst I.C., Doerrer N.G., Hines R.N., Moretto A. (2014). Risk assessment in the 21st century: Roadmap and matrix. Crit. Rev. Toxicol..

[28] Pastoor T.P., Bachman A.N., Bell D.R., Cohen S.M., Dellarco M., Dewhurst I.C., Doe J.E., Doerrer N.G., Embry M.R., Hines R.N. (2014). A 21st century roadmap for human health risk assessment. Crit. Rev. Toxicol..

[29] Simon T.W., Simons S.S., Preston R.J., Boobis A.R., Cohen S.M., Doerrer N.G., Fenner-Crisp P.A., McMullin T.S., McQueen C.A., Rowlands J.C. (2014). The use of mode of action information in risk assessment: Quantitative key events/dose-response framework for modeling the dose-response for key events. Crit. Rev. Toxicol..

[30] Fleck S.C., Churchwell M.I., Doerge D.R., Teeguarden J.G. (2016). Urine and serum biomonitoring of exposure to environmental estrogens II: Soy isoflavones and zearalenone in pregnant women. Food Chem. Toxicol..

[31] Gonzalez L.M., Moeser A.J., Blikslager A.T. (2015). Porcine models of digestive disease: The future of large animal translational research. Transl. Res..

[32] Demaegdt H., Daminet B., Evrard A., Scippo M.-L., Muller M., Pussemier L., Callebaut A., Vandermeiren K. (2016). Endocrine activity of mycotoxins and mycotoxin mixtures. Food Chem. Toxicol..

[33] Patlewicz G., Simon T., Goyak K., Phillips R.D., Rowlands J.C., Seidel S., Becker R.A. (2013). Use and validation of HT/HC assays to support 21st century toxicity evaluations. Regul. Toxicol. Pharmacol..

[34] Gajęcka M., Rybarczyk L., Zwierzchowski W., Jakimiuk E., Zielonka Ł., Obremski K., Gajęcki M. (2011). The effect of experimental, long-term exposure to low-dose zearalenone mycotoxicosis on the histological condition of ovaries in sexually immature gilts. Theriogenology.

[35] Zachariasova M., Dzumana Z., Veprikova Z., Hajkovaa K., Jiru M., Vaclavikova M., Zachariasova A., Pospichalova M., Florian M., Hajslova J. (2014). Occurrence of multiple mycotoxins in European feeding stuffs, assessment of dietary intake by farm animals. Anim. Feed Sci. Technol..

[36] Calabrese E.J. (2005). Paradigm lost, paradigm found: The re-emergence of hormesis as a fundamental dose response model in the toxicological sciences. Environ. Pollut..

[37] Dobrzyński L., Fornalski K.W. Hormesis—Natural phenomenon of answer of organism on stress. Proceeding of the 7th International Scientific Conference on Veterinary Feed Hygiene—The Effects of Mycotoxins on Gastrointestinal Function.

[38] Kramer H.J., van den Ham W.A., Slob W., Pieters M.N. (1996). Conversion factors estimating indicative chronic no-observed-adverse-effect levels from short-term toxicity data. Regul. Toxicol. Pharmacol..

[39] Stopa E., Babińska I., Zielonka Ł., Gajęcki M., Gajęcka M. (2016). Immunohistochemical evaluation of apoptosis and proliferation in the mucous membrane of selected uterine regions in pre-pubertal bitches exposed to low doses of zearalenone. Pol. J. Vet. Sci..

[40] Zielonka Ł., Jakimiuk E., Obremski K., Gajęcka M., Dąbrowski M., Gajęcki M. (2015). An evaluation of the proliferative activity of immunocompetent cells in the jejunal and iliac lymph nodes of prepubertal female wild boars diagnosed with mixed mycotoxicosis. Bull. Vet. Inst. Pulawy.

[41] Vandenberg L.N., Colborn T., Hayes T.B., Heindel J.J., Jacobs D.R., Lee D.-H., Shioda T., Soto A.M., vom Saal F.S., Welshons W.V. (2012). Hormones and endocrine-disrupting chemicals: Low-dose effects and nonmonotonic dose responses. Endocr. Rev..

[42] Grenier B., Applegate T.J. (2013). Modulation of intestinal functions following mycotoxin ingestion: Meta-analysis of published experiments in animals. Toxins.

[43] Hickey G.L., Craig P.S., Luttik R., de Zwart D. (2012). On the quantification of intertest variability in ecotoxicity data with application to species sensitivity distributions. Environ. Toxicol. Chem..

[44] Waśkiewicz A., Beszterda M., Kostecki M., Zielonka Ł., Goliński P., Gajęcki M. (2014). Deoxynivalenol in the gastrointestinal tract of immature gilts under *per os* toxin application. Toxins.

[45] Piotrowska M., Śliżewska K., Nowak A., Zielonka Ł., Żakowska Z., Gajęcka M., Gajęcki M. (2014). The effect of experimental *Fusarium* mycotoxicosis on microbiota diversity in porcine ascending colon contents. Toxins.

[46] Nowak A., Śliżewska K., Gajęcka M., Piotrowska M., Żakowska Z., Zielonka Ł., Gajęcki M. (2015). The genotoxicity of caecal water from gilts following experimentally induced *Fusarium* mycotoxicosis. Vet. Med..

[47] Zielonka Ł., Waśkiewicz A., Beszterda M., Kostecki M., Dąbrowski M., Obremski K., Goliński P., Gajęcki M. (2015). Zearalenone in the intestinal tissues of immature gilts exposed *per os* to mycotoxins. Toxins.

[48] Wan L.Y.M., Turner P.C., El-Nezami H. (2013). Individual and combined cytotoxic effects of *Fusarium* toxins (deoxynivalenol, nivalenol, zearalenone and fumonisins B1) on swine jejunal epithelial cells. Food Chem. Toxicol..

[49] Gajęcki M., Gajęcka M., Zielonka Ł., Jakimiuk E., Obremski K. (2006). Zearalenone as a potential allergen in the alimentary tract—A review. Pol. J. Food Nutr. Sci..

[50] McLaughlin J., Lambert D., Padfield P.J., Burt J.P., O’Neill C.A. (2009). The mycotoxin patulin, modulates tight junctions in caco-2 cells. Toxicol. In Vitro.

[51] Bakhru S.H., Furtado S., Morello A.P., Mathiowitz E. (2013). Oral delivery of proteins by biodegradable nanoparticles. Adv. Drug Deliv. Rev..

[52] Dong M., Tulayakul P., Li J.Y., Dong K.S., Manabe N., Kumagai S. (2010). Metabolic conversion of zearalenone to α-zearalenol by goat tissues. J. Vet. Med. Sci..

[53] Cavret S., Lecoeur S. (2006). Fusariotoxin transfer in animal. Food Chem. Toxicol..

[54] Bracarense A.P., Lucioli J., Grenier B., Pacheco G.D., Moll W.D., Schatzmayr G., Oswald I.P. (2012). Chronic ingestion of deoxynivalenol and fumonisin, alone or in interaction, induces morphological and immunological changes in the intestine of piglets. Br. J. Nutr..

[55] Diczfalusy U., Bjorkhem I. (2011). Still another activity by the highly promiscuous enzyme CYP3A4: 25-hydroxylation of cholesterol. J. Lipid Res..

[56] Hulce J.J., Cognetta A.B., Niphakis M.J., Tully S.E., Cravatt B.F. (2013). Proteome-wide mapping of cholesterol-interacting proteins in mammalian cells. Nat. Methods.

[57] Lathe R., Kotelevtsev Y. (2014). Steroid signaling: Ligand-binding promiscuity molecular symmetry, and the need for gating. Steroids.

[58] Gajęcka M., Otrocka-Domagała I. (2013). Immunocytochemical expression of 3β- and 17β-hydroxysteroid dehydrogenase in bitch ovaries exposed to low doses of zearalenone. Pol. J. Vet. Sci..

[59] Maćkowiak B., Wang H. (2016). Mechanisms of xenobiotic receptor activation: Direct vs. indirect. BBA Gene Regul. Mech..

[60] Brzuzan P., Woźny M., Wolińska L., Piasecka A., Florczyk M., Jakimiuk E., Góra M., Łuczyński M.K., Gajęcki M. (2015). MicroRNA expression profiles in liver and colon of sexually immature gilts after exposure to *Fusarium* mycotoxins. Pol. J. Vet. Sci..

[61] Tawfik D.S. (2010). Messy biology and the origins of evolutionary innovations. Nat. Chem. Biol..

[62] Weng J.K., Noel J.P. (2012). The remarkable pliability and promiscuity of specialized metabolism. Cold Spring Harb. Symp. Quant. Biol..

[63] Zielonka Ł., Gajęcka M., Rozicka A., Dąbrowski M., Żmudzki J., Gajęcki M. (2014). The Effect of environmental mycotoxins on selected ovarian tissue fragments of multiparous female wild boars at the beginning of astronomical winter. Toxicon.

[64] Kradolfer D., Flöter V.L., Bick J.T., Fürst R.W., Rode K., Brehm R., Henning H., Waberski D., Bauersachs S., Ulbrich S.E. (2016). Epigenetic effects of prenatal estradiol-17b exposure on the reproductive system of pigs. Mol. Cell. Endocrinol..

[65] Luu-The V. (2013). Assessment of steroidogenesis and steroidogenic enzyme functions. J. Steroid Biochem..

[66] Goldstone J.V., Sundaramoorthy M., Zhao B., Waterman M.R., Stegeman J.J., Lamb D.C. (2016). Genetic and structural analyses of cytochrome P450 hydroxylases in sex hormone biosynthesis: Sequential origin and subsequent coevolution. Mol. Phylogenet. Evol..

[67] Warmerdam E.G., Visser M., Coelingh Bennink H.J., Groen M. (2008). A new route of synthesis of estetrol. Climacteric.

[68] Aidoo-Gyamfi K., Cartledge T., Shah K., Ahmed S. (2009). Estrone sulfatase and its inhibitors. Anticancer Agents Med. Chem..

[69] Bondesson M., Hao R., Lin C.Y., Williams C., Gustafsson J.-Å. (2015). Estrogen receptor signaling during vertebrate development. BBA Gene Regul. Mech..

[70] Arlt W., Martens J.W., Song M., Wang J.T., Auchus R.J., Miller W.L. (2002). Molecular evolution of adrenarche: Structural and functional analysis of p450c17 from four primate species. Endocrinology.

[71] Greaves R.F., Jevalikar G., Hewitt J.K., Zacharin M.R. (2014). A guide to understanding the steroid pathway: New insights and diagnostic implications. Clin. Biochem..

[72] Snawder J.E., Lipscomb J.C. (2000). Interindividual variance of cytochrome P450 forms in human hepatic microsomes: Correlation of individual forms with xenobiotics metabolism and implications in risk assessment. Regul. Toxicol. Pharmacol..

[73] Gajęcka M., Jakimiuk E., Zielonka Ł., Obremski K., Gajęcki M. (2009). The biotransformation of chosen mycotoxins. Pol. J. Vet. Sci..

[74] Sayers E.W., Barrett T., Benson D.A., Bolton E., Bryant S.H., Canese K., Chetvernin V., Church D.M., Dicuccio M., Federhen S. (2012). Database resources of the National Center for Biotechnology Information. Nucleic Acids Res..

[75] Kisiela M., Skarka A., Ebert B., Maser E. (2012). Hydroxysteroid dehydrogenases (HSDs) in bacteria—A bioinformatic perspective. J. Steroid Biochem..

[76] Payne D.W., Talalay P. (1985). Isolation of novel microbial 3α-, 3β-, and 17-β hydroxysteroid dehydorgenases. J. Biol. Chem..

[77] Drasar B.S., Hill M.J. (1972). Intestinal bacteria and cancer. Am. J. Clin. Nutr..

[78] Clark D.T., Soory M. (2006). The metabolism of cholesterol and certain hormonal steroids by Treponema denticola. Steroids.

[79] Frizzell C., Ndossi D., Verhaegen S., Dahl E., Eriksen G., Sørlie M., Ropstad E., Muller M., Elliott C.T., Connolly L. (2011). Endocrine disrupting effects of zearalenone, alpha- and beta-zearalenol at the level of nuclear receptor binding and steroidogenesis. Toxicol. Lett..

[80] Endo S., Miyagi N., Matsunaga T., Hara A., Ikari A. (2016). Human dehydrogenase/reductase (SDR family) member 11 is a novel type of 17β-hydroxysteroid dehydrogenase. Biochem. Biophys. Res. Commun..

[81] Hueza I.M., Raspantini P.C.F., Raspantini L.E.R., Latorre A.O., Górniak S.L. (2014). Zearalenone, an estrogenic mycotoxin, is an immunotoxic compound. Toxins.

[82] Marchais-Oberwinkler S., Henn C., Moller G., Klein T., Negri M., Oster A., Spadaro A., Werth R., Wetzel M., Xu K. (2011). 17β-Hydroxysteroid dehydrogenases (17β-HSDs) as therapeutic targets: Protein structures, functions, and recent progress in inhibitor development. J. Steroid Biochem..

[83] Huhtinen K., Stahle M., Perheentupa A., Poutanen M. (2012). Estrogen biosynthesis and signaling in endometriosis. Mol. Cell Endocrinol..

[84] Rajaram R.D., Brisken C. (2012). Paracrine signaling by progesterone. Mol. Cell Endocrinol..

[85] Gajęcki M., Gajęcka M., Jakimiuk E., Zielonka L., Obremski K., Rai M., Ajit V. (2010). Zearalenone—Undesirable substance. Mycotoxins in Food, Feed and Bioweapons.

[86] Gajęcka M. (2012). The effect of low-dose experimental zearalenone intoxication on the immunoexpression of estrogen receptors in the ovaries of pre-pubertal bitches. Pol. J. Vet. Sci..

[87] Warnmark A., Treuter E., Wright A.P., Gustafsson J.Å. (2003). Activation functions 1 and 2 of nuclear receptors: Molecular strategies for transcriptional activation. Mol. Endocrinol..

[88] López-Calderero I., Carnero A., Astudillo A., Palacios J., Chaves M., Benavent M., Limón M.L., Garcia-Carbonero R. (2014). Prognostic relevance of estrogen receptor-α Ser167 phosphorylation in stage II-III colon cancer patients. Hum. Pathol..

[89] Zuloaga D.G., Zuloaga K.L., Hinds L.R., Carbone D.L., Handa R.J. (2014). Estrogen receptor β expression in the mouse forebrain: Age and sex differences. J. Comp. Neurol..

[90] Wu W.F., Tan X.J., Dai Y.B., Krishnan V., Warner M., Gustafsson J.Å. (2013). Targeting estrogen receptor b in microglia and T cells to treat experimental autoimmune encephalomyelitis. Proc. Natl. Acad. Sci. USA.

[91] Warner M., Gustafsson J.-A. (2015). DHEA—A precursor of ERβ ligands. J. Steroid Biochem..

[92] Wada-Hiraike O., Imamov O., Hiraike H., Hultenby K., Schwend T., Omoto Y., Warner M., Gustafsson J.Å. (2006). Role of estrogen receptor beta in colonic *Epithelium*. Proc. Natl. Acad. Sci. USA.

[93] Papoutsi Z., Zhao C., Putnik M., Gustafsson J., Dahlman-Wright K. (2009). Binding of estrogen receptor alpha/beta heterodimers to chromatin in MCF-7 cells. J. Mol. Endocrinol..

[94] Takemura H., Shim J.Y., Sayama K., Tsubura A., Zhu B.T., Shimoi K. (2007). Characterization of the estrogenic activities of zearalenone and zeranol in vivo and in vitro. J. Steroid Biochem..

[95] Mueller S., Simon S., Chae K., Metzler M., Korach K.S. (2004). Phytoestrogens and their human metabolites show distinct agonistic and antagonistic properties on estrogen receptor α (ERa) and ERβ in human cells. Toxicol. Sci..

[96] Levin E.R., Pietras R.J. (2008). Estrogen receptors outside the nucleus in breast cancer. Breast Cancer Res. Treat..

[97] Barton M. (2012). Position paper: The membrane estrogen receptor GPER—Clues and questi. Steroids.

[98] Barton M. (2016). Not lost in translation: Emerging clinical importance of the G protein-coupled estrogen receptor GPER. Review Article. Steroids.

[99] Bodiga V.L., Boindala S., Putcha U., Subramaniam K., Manchala R. (2005). Chronic low intake of protein or vitamins increases the intestinal epithelial cell apoptosis in Wistar/NIN rats. Nutrition.

[100] Marin D.E., Taranu I., Burlacu R., Manda G., Motiu M., Neagoe I., Dragomir C., Stancu M., Calin L. (2011). Effects of zearalenone and its derivatives on porcine immune response. Toxicol. In Vitro.

[101] Solhaug A., Karlsøen L.M., Holme J.A., Kristoffersen A.B., Eriksen G.S. (2016). Immunomodulatory effects of individual and combined mycotoxins in the THP-1 cell line. Toxicol. In Vitro.

[102] Obremski K. (2014). The effect of in vivo exposure to zearalenone on cytokine secretion by Th1 and Th2 lymphocytes in porcine Peyer’s patches after in vitro stimulation with LPS. Pol. J. Vet. Sci..

[103] Bryden W.L. (2012). Mycotoxin contamination of the feed supply chain: Implications for animal productivity and feed security. Anim. Feed Sci. Technol..

[104] Knudsen K.E.B., Lærke H.N., Ingerslev A.K., Hedemann M.S., Nielsen T.S., Theil P.K. (2016). Carbohydrates in pig nutrition—Recent advances. J. Anim. Sci..

[105] Lupescu A., Bissinger R., Jilani K., Lang F. (2014). In vitro induction of erythrocyte phosphatidyloserine translocation by the natural Naphthoquinone Shikonin. Toxins.

[106] Avantaggiato G., Havenaar R., Visconti A. (2004). Evaluation of the intestinal absorption of deoxynivalenol and nivalenol by an in vitro gastrointestinal model, and the binding efficacy of activated carbon and other adsorbent materials. Food Chem. Toxicol..

[107] Maresca M. (2013). From the gut to the brain: Journey and pathophysiological effects of the food-associated trichothecene mycotoxin deoxynivalenol. Toxins.

[108] Kollarczik B., Gareis M., Hanelt M. (1994). In vitro transformation of the Fusarium mycotoxins deoxynivalenol and zearalenone by the normal gut microflora of pigs. Nat. Toxins.

[109] Gajęcka M., Stopa E., Tarasiuk M., Zielonka Ł., Gajęcki M. (2013). The expression of type-1 and type-2 nitric oxide synthase in selected tissues of the gastrointestinal tract during mixed mycotoxicosis. Toxins.

[110] Lucioli J., Pinton P., Callu P., Laffitte J., Grosjean F., Kolf-Clauw M., Oswald I.P., Bracarense A.P. (2013). The food contaminant deoxynivalenol activates the mitogen activated protein kinases in the intestine: Interest of ex vivo models as an alternative to in vivo experiments. Toxicon.

[111] Sergent T., Ribonnet L., Kolosova A., Garsou S., Schaut A., de Saeger S., van Peteghem C., Larondelle Y., Pussemier L., Schneider Y.J. (2008). Molecular and cellular effects of food contaminants and secondary plant components and their plausible interactions at the intestinal level. Food Chem.Toxicol..

[112] Cutolo M., Sulli A., Straub R.H. (2012). Estrogen metabolism and autoimmunity. Autoimmun. Rev..

[113] Chen F., Lin P., Wang N., Yang D., Wen X., Zhou D., Wang A., Jin Y. (2016). Herp depletion inhibits zearalenone-induced cell death in RAW 264.7 macrophages. Toxicol. In Vitro.

[114] Silva-Campa E., Mata-Haro V., Mateu E., Hernández J. (2012). Porcine reproductive and respiratory syndrome virus induces CD4^+^CD8^+^CD25^+^Foxp3^+^ regulatory T cells (Tregs). Virology.

[115] Yan X.-J., Feng C.-C., Liu Q., Zhang L.-Y., Dong X., Liu Z.-L., Cao Z.-J., Mo J.-Z., Li Y., Fang J.-Y. (2014). Vagal afferents mediate antinociception of estrogen in a rat model of visceral pain: The involvement of intestinal mucosal mast cells and 5-hydroxytryptamine 3 signaling. J. Pain.

[116] Taylor S.E., Martin-Hirsch P.L., Martin F.L. (2010). Oestrogen receptor splice variants in the pathogenesis of disease. Cancer Lett..

[117] Thomas C., Gustafsson J.Å. (2011). The different roles of ER subtypes in cancer biology and therapy. Nat. Rev. Cancer.

[118] Oduwole O.O., Isomaa V.V., Nokelainen P.A., Stenback F., Vihko P.T. (2002). Down regulation of estrogen-metabolizing 17 beta-hydroxysteroid dehydrogenase type 2 expression correlates inversely with Ki67 proliferation marker in colon-cancer development. Int. J. Cancer.

[119] Gajęcka M., Woźny M., Brzuzan P., Zielonka Ł., Gajęcki M. (2011). Expression of CYPscc and 3β-HSD mRNA in bitches ovary after long-term exposure to zearalenone. Bull. Vet. Inst. Pulawy.

[120] Juengel J.L., Heath D.A., Quirke L.D., McNatty K.P. (2006). Oestrogen receptor α and β, androgen receptor and progesterone receptor mRNA and protein localization within the developing ovary and in small growing follicles of sheep. Reproduction.

[121] Bishop C.V., Stormshak F. (2008). Non-genomic actions of progesterone and estrogens in regulating reproductive events in domestic animals. Vet. J..

[122] Richards J.D., Gong J., de Lange C.F.M. (2005). The gastrointestinal microbiota and its role in monogastric nutrition and health with an emphasis on pigs: Current understanding, possible modulations, and new technologies for ecological studies. Can. J. Anim. Sci..

[123] Culpepper T., Mai V. (2013). Evidence for contributions of gut microbiota to colorectal carcinogenesis. Curr. Nutr. Rep..

[124] Broom L. (2015). Mycotoxins and the intestine. Anim. Nutr..

[125] Pedersen K., Tannock G.W. (1989). Colonization of the porcine gastrointestinal-tract by lactobacilli. Appl. Environ. Microbiol..

[126] Giang H.H., Viet T.Q., Ogle B., Lindberg J.E. (2010). Growth performance, digestibility, gut environment and health status in weaned piglets fed a diet supplemented with potentially probiotic complexes of lactic acid bacteria. Livest. Sci..

[127] Rovers M. (2012). Healthy pigs with less use of antibiotics—A nutritional approach in three steps. Int. Pigs Top..

[128] Franco T.S., Garcia S., Hirooka E.Y., Ono Y.S., dos Santos J.S. (2011). Lactic acid bacteria in the inhibition of *Fusarium graminearum* and deoxynivalenol detoxification. J. Appl. Microbiol..

[129] El-Nezami H.S., Chrevatidis A., Auriola S., Salminen S., Mykkanen H. (2002). Removal of common *Fusarium* toxins in vitro by strains of *Lactobacillus* and *Propionibacterium*. Food Addit. Contam..

[130] Burel C., Tanguy M., Guerre P., Boilletot E., Cariolet R., Queguiner M., Postollec G., Pinton P., Salvat G., Oswald I.P. (2013). Effect of low dose of fumonisins on pig health: Immune status, intestinal microbiota and sensitivity to *Salmonella*. Toxins.

[131] Drew M.D., van Kessel A.G., Estrada A.E., Ekpe E.D., Zijlstra R.T. (2002). Effect of dietary cereal on intestinal bacterial populations in weaned pigs. Can. J. Anim. Sci..

[132] Hughes R., Rowland I.R. (2000). Metabolic activities of the gut microflora in relation to cancer. Microb. Ecol. Health Dis..

[133] De Moreno de LeBlanc A., Perdigón G. (2005). Reduction of β-glukuronidase and reductase activity by yoghurt in a murine colon cancer model. Biocell.

[134] Macfarlane G.T., Macfarlane S. (2007). Models for intestinal fermentation: Association between food components, delivery systems, bioavailability and functional interactions in the gut. Curr. Opin. Biotechnol..

[135] Zinedine A., Soriano J.M., Molto J.C., Manes J. (2007). Review on the toxicity, occurrence, metabolism, detoxification, regulations and intake of zearalenone: An oestrogenic mycotoxin. Food Chem. Toxicol..

[136] Kuciel-Lisieska G., Obremski K., Stelmachów J., Gajęcka M., Zielonka Ł., Jakimiuk E., Gajęcki M. (2008). Presence of zearalenone in blood plasma in women with neoplastic lesions in the mammary gland. Bull. Vet. Inst. Pulawy.

[137] Klinder A., Karlsson P.C., Clune Y., Hughes R., Glei M., Rafter J., Rowland I., Collins J.K., Pool-Zobel B.L. (2007). Faecal water as a non-invasive biomarker in nutritional intervention: Comparison of preparation methods and refinement of different endpoints. Nutr. Cancer.

[138] Pearson J.R., Gill C.I.R., Rowland I.R. (2009). Diet, faecal water, and colon cancer—Development of a biomarker. Nutr. Rev..

[139] De Ruyck K., de Bovre M., Huybrechts I., de Saeger S. (2015). Dietary mycotoxins, co-exposure, and carcinogenesis in humans: Short review. Mutat. Res. Rev. Mutat. Res..

[140] Van der Aar P.J., Molist F., van der Klis J.D. (2016). The central role of intestinal health on the effectof feed additives on feed intake in swine and poultry. Anim. Feed.

[141] Antonissen G., Martel A., Pasmans F., Ducatelle R., Verbrugghe E., Vandenbroucke V., Li S., Haesebrouck F., Immerseel F.V., Croubels S. (2014). The impact of Fusarium mycotoxins on human and animal host susceptibility to infectious diseases. Toxins.

[142] Grześk E., Grześk G., Koziński M., Stolarek W., Zieliński M., Kubica J. (2011). Nitric oxide as a cause and a potential place therapeutic intervention in hyporesponsiveness vascular in early sepsis. Folia Cardiol..

[143] Bennett M.R. (1997). Non-adrenergic non-cholinergic (NANC) transmission to smooth muscle: 35 years on. Prog. Neurobiol..

[144] Carlson S.J., Chang M.I., Nandivada P., Cowan E., Puder M. (2013). Neonatal intestinal physiology and failure. Semin. Pediatr. Surg..

[145] Dijkstra G., van Goor H., Jansen P.L., Moshage H. (2004). Targeting nitric oxide in the gastrointestinal tract. Curr. Opin. Investig. Drugs.

[146] Davila A.-M., Blachier F., Gotteland M., Andriamihaja M., Benetti P.-H., Sanz Y., Tomé D. (2013). Re-print of “Intestinal luminal nitrogen metabolism: Role of the gut microbiota and consequences for the host”. Pharmacol. Res..

[147] Yang E.J., Yim E.Y., Song G., Kim G.O., Hyun C.G. (2009). Inhibition of nitric oxide production in lipopolysaccharide-activated RAW 264.7 macrophages by Jeju plant extracts. Interdiscip. Toxicol..

[148] Prodanov-Radulović J., Došenz R., Stojanov I., Polaček V., Živkov-Baloš M., Marčić D., Pušić I. (2014). The interaction between the swine infectious diseases agents and low levels of mycotoxins in swine feed. Biotechnol. Anim. Husb..

[149] Maresca M., Yahi N., Younès-Sakr L., Boyron M., Caporiccio B., Fantini J. (2008). Both direct and indirect effects account for the proinflammatory activity of enteropathogenic mycotoxins on the human intestinal epithelium: Stimulation of interleukin-8 secretion, potentiation of interleukin-1beta effect and increase in the transepithelial passage of commensal bacteria. Toxicol. Appl. Pharmacol..

[150] Levkut M., Revajova V., Slaminkova Z., Levkutova M., Borutova R., Gresakova L., Leng L. (2011). Lymphocyte subpopulations in blood and duodenal epithelium of broilers fed diets contaminated with deoxynivalenol and zearalenone. Anim. Feed Sci. Technol..

[151] Prims S., Tambuyzer B., Vergauwen H., Huygelen V., van Cruchten S., van Ginneken C., Casteleyn C. (2016). Intestinal immune cell quantification and gram type classification of the adherent microbiota in conventionally and artificially reared, normal and low birth weight piglets. Livest. Sci..

[152] Gajęcka M., Tarasiuk M., Zielonka Ł., Dąbrowski M., Gajęcki M. (2016). Risk assessment for changes in metabolic profile and body weight of pre-pubertal gilts during long-term monotonic exposure to low doses of zearalenone (ZEN). Res. Vet. Sci..

[153] Jilani K., Lang F. (2013). Ca^2+^-dependent suicidal erythrocyte death following zearalenone exposure. Arch. Toxicol..

[154] Jiang S.Z., Yang Z.B., Yang W.R., Gao J., Liu F.X., Broomhead J., Chi F. (2011). Effects of purified zearalenone on growth performance, organ size, serum metabolites, and oxidative stress in postweaning gilts. J. Anim. Sci..

[155] Ingawale D.K., Mandlikb S.K., Naik S.R. (2014). Models of hepatotoxicity and the underlying cellular, biochemical and immunological mechanism(s): A critical discussion. Environ. Toxicol. Pharmacol..

[156] Gajęcka M., Rybarczyk L., Jakimiuk E., Zielonka Ł., Obremski K., Zwierzchowski W., Gajęcki M. (2012). The effect of experimental long-term exposure to low-dose zearalenone on uterine histology in sexually immature gilts. Exp. Toxicol. Pathol..

[157] Mendonça F.M., de Sousa F.R., Barbosa A.L., Martins S.C., Araújo R.L., Soares R., Abreu C. (2015). Metabolic syndrome and risk of cancer: Which link?. Metabolism.

[158] Jiang S.Z., Yang Z.B., Yang W.R., Wang S.J., Liu F.X., Johnston L.A., Chi F., Wang Y. (2012). Effect of purified zearalenone with or without modified montmorillonite on nutrient availability, genital organs and serum hormones in post-weaning piglets. Livest. Sci..

[159] Liu Q., Wang Y., Gu J., Yuan Y., Liu X., Zheng W., Huang Q., Liu Z., Bian J. (2014). Zearalenone inhibits testosterone biosynthesis in mouse Leydig cells via the crosstalk of estrogen receptor signaling and orphan nuclear receptor Nur77 expression. Toxicol. In Vitro.

[160] Tiemann U., Brüssow K.-P., Küchenmeister U., Jonas L., Kohlschein P., Pöhland R., Dänicke S. (2006). Influence of diets with cereal grains contaminated by graded levels of two *Fusarium* toxins on selected enzymatic and histological parameters of liver in gilts. Food Chem. Toxicol..

[161] Serrano A.B., Meca G., Font G., Ferrer E. (2014). Risk assessment of beauvericin, enniatins and fusaproliferin present in follow-up infant formula by in vitro evaluation of the duodenal and colonic bioaccessibility. Food Control.

[162] Wang J., Ji H.F., Hou C.L., Wang S.X., Zhang D.Y., Liu H., Shan D.C., Wang Y.M. (2014). Effects of *Lactobacillus johnsonii* XS4 supplementation on reproductive performance, gut environment, and blood biochemical and immunological index in lactating sows. Livest. Sci..

[163] De Angelis E., Monaci L., Visconti A. (2014). Investigation on the stability of deoxynivalenol and DON-3 glucoside during gastro-duodenal in vitro digestion of a naturally contaminated bread model food. Food Control.

[164] Etzel R.A. (2006). What the primary care pediatrician should know about syndromes associated with exposures to mycotoxins. Curr. Probl. Pediatr. Adolesc. Health Care.

[165] Fleck S.C., Hildebrand A.A., Müller E., Pfeiffer E., Metzler M. (2012). Genotoxicity and inactivation of catechol metabolites of the mycotoxin zearalenone. Mycotoxin Res..

[166] Büning C., Geissler N., Prager M., Sturm A., Baumgart D.C., Büttner J., Bühner S., Haas V., Lochs H. (2012). Increased small intestinal permeability in ulcerative colitis: Rather genetic than environmental and a risk factor for extensive disease?. Inflamm. Bowel Dis..

[167] Gerez J.R., Pinton P., Callu P., Grosjean F., Oswald I.P., Bracarense A.P.F.L. (2015). Deoxynivalenol alone or in combination with nivalenol and zearalenone induce systemic histological changes in pigs. Exp. Toxicol. Pathol..

[168] Speranda M., Liker B., Speranda T., Seric V., Antunovic Z., Grabarevic Z., Sencic D., Grguric D., Steiner Z. (2006). Haematological and biochemical parameters of weaned piglets fed on fodder mixture contaminated by zearalenone with addition of clinoptilolite. Acta Vet. Beograd..

[169] Jakimiuk E., Kuciel-Lisiecka G., Zwierzchowski W., Gajęcka M., Obremski K., Zielonka Ł., Skorska-Wyszyńska E., Gajęcki M. (2006). Morphometric changes of the reproductive system in gilts during zearalenone mycotoxicosis. Med. Weter..

[170] De Boevre M., Graniczkowska K., de Saeger S. (2015). Metabolism of modified mycotoxins studied through in vitro and in vivo models: An overview. Toxicol. Lett..

[171] Parr T., Mareko M.H.D., Ryan K.J.P., Hemmings K.M., Brown D.M., Brameld J.M. (2016). The impact of growth promoters on muscle growth and the potential consequences for meat quality. Meat Sci..

[172] Zhabinskii V.N., Khripach N.B., Khripach V.A. (2015). Steroid plant hormones: Effects outside plant kingdom. Steroids.

